# Dexmedetomidine Attenuates Myocardial Ischemia-Reperfusion Injury in Diabetes Mellitus by Inhibiting Endoplasmic Reticulum Stress

**DOI:** 10.1155/2019/7869318

**Published:** 2019-11-30

**Authors:** Jinjie Li, Ying Zhao, Nan Zhou, Longyun Li, Kai Li

**Affiliations:** ^1^Department of Anesthesiology, China-Japan Union Hospital of Jilin University, No. 126, Xiantai Street, Changchun, Jilin 130033, China; ^2^Department of Nephrology, The First Hospital of Jilin University, No. 71, Xinmin Street, Changchun Jilin 130021, China; ^3^Department of Anesthesia, The General Hospital of Northern Theater Command, Shenyang 110016, China

## Abstract

**Objective:**

With the increasing incidence of diabetes mellitus (DM) combined with myocardial ischemia, how to reduce myocardial ischemia-reperfusion injury in DM patients has become a major problem faced by clinicians. We investigated the therapeutic effects of dexmedetomidine (DEX) on myocardial ischemia-reperfusion injury in DM rats and its effect on endoplasmic reticulum stress.

**Methods:**

SD rats with SPF grade were randomly divided into 6 groups: non-DM rats were divided into the sham operation group (NDM-S group), ischemia-reperfusion group (NDM-IR group), and dexmedetomidine group (NDM-DEX group); DM rats were divided into the diabetic sham operation group (DM-S group), diabetes-reperfusion group (DM-IR group), and diabetes-dexmedetomidine (DM-DEX) group, with 10 rats in each group. Then the effects of DEX on the changes of CK-MB and cTnT levels were examined. The effects of myocardial pathological damage and myocardial infarct size were detected. The apoptosis of cardiomyocytes was detected. The apoptosis of heart tissue cells was also tested through the expressions of cleaved caspase-3, Bcl-2, and Bax proteins. The expression of endoplasmic reticulum stress-related proteins GRP78, CHOP, ERO1*α*, ERO1*β*, and PDI was examined. The hypoxia/reoxygenation (H/R) injury cell model was established, the effects of DEX, DEX+ ERS agonist on cell apoptosis was also detected.

**Results:**

The myocardial damage of DM-IR was more severe than that of NDM-IR rats. DEX could reduce the expression of CK-MB and cTnT, reduce pathological damage, and reduce scar formation and improve fibrosis. DEX can reduce the expression of GRP78, CHOP, ERO1*α*, ERO1*β*, and PDI proteins in vivo and in vitro. And the effect of DEX on cell apoptosis could be blocked by ERS agonist.

**Conclusion:**

DEX attenuates myocardial ischemia-reperfusion injury in DM rats and H/R injury cell, which is associated with the reduction of ERS-induced cardiomyocyte apoptosis.

## 1. Introduction

Cardiovascular disease is listed as one of the greatest health threats to humans in the 21st century. The incidence and mortality of cardiovascular diseases in China have been increasing continuously. There are about 230 million people with cardiovascular disease in the country, and about 3 million people die every year from cardiovascular diseases [1]. Ischemic heart disease (IHD) is one of the most important diseases in cardiovascular disease, and it is also one of the most harmful diseases that seriously affect the survival rate and quality of life of patients. In developing countries, the incidence rate of ischemic heart disease is rising.

Myocardial ischemia-reperfusion is the main treatment for myocardial ischemia. Although it can bring about the oxygen and nutrients needed for myocardial aerobic metabolism, it also brings more serious myocardial damage to the myocardium called perfusion injury (MI/RI) [2]. Therefore, how to choose appropriate drugs and methods to prevent and avoid MI/RI and the search for effective myocardial protection measures have become one of major clinical problems.

Diabetes (DM) is a chronic metabolic disease characterized by the inability to maintain a balance of blood glucose levels. The high incidence of DM has received worldwide attention. According to WHO estimates, there will be approximately 360 million DM patients worldwide in 2025 [3]. The risk of IHD in patients with DM is higher than that in patients with non-DM. Compared with patients with normal blood glucose, the risk of death from DM patients with IHD is twice as high as in normal controls [4]. Therefore, how to reduce MI/RI in DM patients has become a major problem for clinicians.

More and more studies have confirmed that endoplasmic reticulum stress is closely related to the occurrence and development of cardiovascular diseases such as atherosclerosis and myocardial ischemia [5, 6]. The endoplasmic reticulum (ER) is an important organelle present in many eukaryotic cells, which is responsible for ectopic intracellular protein folding, posttranscriptional modification (including glycosylation and formation of disulfide bonds), lipid synthesis, and Ca^2+^ storage [7, 8]. Under the stimulation of high glucose, ischemia and hypoxia, and Ca^2+^ homeostasis, the ER internal balance is disrupted, resulting in accumulation of unfolded or misfolded proteins in the cell, which leads to endoplasmic reticulum stress (ERS) [9, 10]. Once ERS presents too long, it induces the expression of apoptotic signaling pathways inducing ultimate apoptosis [11, 12].

It has been proved that ERS is involved in islet *β*-cell failure and is closely related to the occurrence of DM. Hyperglycemia also causes excessive production of reactive oxygen species, which in turn activates the ERS pathway and participates in the apoptosis process [13]. Activation of C/EBP homologous protein (CHOP) is the main pathway for apoptosis in ERS downstream. CHOP is an ERS-specific nuclear transcription factor, which acts as an endoplasmic reticulum-associated proapoptotic protein. CHOP expresses extremely low under normal conditions; however, when ERS occurs, it is induced to be expressed in a large amount and is transferred to the nucleus to regulate its target genes Ero1*α* and Bcl-2 and induce apoptosis [14, 15]. With the deepening of ERS awareness, how to effectively prevent and treat ERS-mediated apoptosis has become an important part of DM and myocardial ischemia research.

Dexmedetomidine (DEX) is a highly selective *α*2 adrenergic receptor agonist with a good sedative effect and no respiratory depression. It is a commonly used sedative analgesic in patients [16]. Studies have found that DEX has a protective effect on ischemia-reperfusion injury of the heart, kidney, and other organs and can alleviate cerebral ischemia-reperfusion injury in DM rats by inhibiting oxidative stress and inflammatory response [17–19]. However, the therapeutic effect on an ERS rat myocardial ischemia reperfusion injury model and its effect on ERS have not been reported.

This experiment intends to establish a type 2 DM rat model and an H/R cell model to observe the therapeutic effect of DEX posttreatment on myocardial ischemia-reperfusion injury and analyze the relationship between DEX and ERS.

## 2. Materials and Methods

### 2.1. Material and Drug

Dexmedetomidine hydrochloride (molecular weight 236.74, purity greater than 99%, batch no.100523-201301) was purchased from National Institutes for Food and Drug Control in China, dissolved in ddH_2_O, prepared into 1 mg/mL solution, and stored at 4°C. Streptozotocin (STZ, molecular weight 265.22, purity greater than 98%, lot no. 18883-66-4) was purchased from Sigma. The STZ powder was dissolved in 0.1 mmol/L citrate buffer and configured as a 1% STZ solution [20].

### 2.2. Rat Model Preparation, Grouping, and Treatment

#### 2.2.1. Preparation of Type 2 Diabetes Rat Model

Eight-week-old SPF male Sprague-Dawley rats weighing 200-250 g were housed in cages (fed freely and drink water), maintained at 20-25°C at room temperature (40-60% humidity), and exposed to light for 12 hours a day. One week after rat rearing, a rat type 2 DM model was established according to literature reports [21, 22]. Rats with type 2 DM were fed high-fat diet for 4 weeks. Rats were fasted for 12 h before model establishment. After intraperitoneal injection of 1% streptozotocin (35 mg/kg) for one week, the blood glucose of tail venous was measured. When the content of Glu ≥ 16.7 mmol/L, with typical DM symptoms such as polydipsia, polyphagia, and polyuria present, the DM rat models were established successfully. Then rats were fed high-fat diet and randomly assigned to the DM group after 4 weeks. The blood glucose was remeasured to make sure Glu ≥ 16.7 mmol/L before the experiment. Non-DM rats were fed with normal food, and the same dose of sodium citrate buffer was intraperitoneally used as the control group.

#### 2.2.2. Animal Grouping

The rats were randomly divided into 6 groups: non-DM rats were divided into the sham operation group (NDM-S group), ischemia-reperfusion group (NDM-IR group), dexmedetomidine group (NDM-DEX group); DM rats were divided into the diabetic sham operation group (DM-S group), diabetes-reperfusion group (DM-IR group), diabetes-dexmedetomidine (DM-DEX) group, with 10 rats in each group only.

#### 2.2.3. Preparation of Rat Model of Ischemia-Reperfusion Injury

The rat model of ischemia-reperfusion injury was prepared according to a previous method [23, 24]. Rats were given anesthesia with 1% pentobarbital sodium, and then the electrodes were inserted into the rat's limb to record the II lead electrocardiogram. After tracheotomy, rats were connected with a respiratory machine (tidal volume 2.5 mL, respiratory rate 70-80 min). The carotid artery was intubated and connected to a ventilator to record changes in heart rate and blood pressure. The femoral vein indwelling catheter is used to supplement saline during surgery. After thoracotomy, the injury was performed at a distance of 4 mm from the root of the left anterior descending (LAD) coronary artery for 30 min. The electrocardiogram showed that the ST segment was uplifted upward, which was successful for myocardial ischemia and unrestrained. Reperfusion was maintained for 120 min, resulting in reperfusion. The DEX group was intravenously injected with 7.5 *μ*g/kg DEX at a rate of 5 *μ*g/kg/h, 30 min before surgery according to a reference with a little modification [25]. The other groups were injected with the same amount of normal saline.

### 2.3. CK-MB and cTnT Were Detected by ELISA Assay

At 120 min of reperfusion, blood was collected from the abdominal aorta, centrifuged at 10,000 rpm for 10 min at 4°C. The supernatant was taken, and CK-MB and troponin (cTnT) in the serum were measured by ELISA according to the kit instructions. The microplate reader detects the absorbance at 450 nm, and the standard curve is drawn according to the OD value of the gradient concentration standard. The regression equation is obtained, and CK-MB and cTnT in the serum are calculated according to the regression equation.

### 2.4. Pathological Examination

After the arterial blood was collected, the rats were euthanized, the heart was quickly removed, the lung tissue was fixed with 4% paraformaldehyde, dehydrated to transparency, paraffin-embedded and then sliced to a thickness of about 5 *μ*m, and dried in a constant temperature oven at 40°C. In xylene dewaxing, gradual ethanol solution hydration, and Harris hematoxylin dyeing solution for 5 min, the slices were differentiated by l% hydrochloric acid, washed by 1% ammonia water (5-10 s), dyed with eosin stain (30-60 s), dehydrated to transparency, and sealed by medium gum. Histological changes were observed under a light microscope.

### 2.5. Masson Trichrome Staining

Masson's Trichrome Stain Kit (G1340, Solarbio, Beijing, China), 4 *μ*m thick paraffin sections, hematoxylin staining for 1 min, deionized water washing for 30 s, eosin staining for 40 s, deionized water washing for 30 s, aniline blue staining for 2.5 min, deionized water washing for 10 s, dehydrating by 100% ethanol for 30 s and washing by xylene for 15 min, and sealing with resinous mounting medium (G8590, Solarbio) were used. Finally, we observed sections with a light microscope. The infarct area of each heart slice and the entire left ventricular area were analyzed using Image-Pro Plus software (Media Cybernetics Inc., Carlsbad, CA, USA). The infarct size was calculated according to the following formula: infarct size (%) = (myocardial infarct size/total left ventricular area) × 100%.

### 2.6. TUNEL Assay

Each group of myocardial tissue sections was taken and operated according to the TUNEL Apoptosis Detection Kit. Six fields of view were randomly selected under each slice microscope to observe apoptotic cells in the field of view and count. The percentage of apoptotic cells in the statistical field is a percentage of the total number of cardiomyocytes.

### 2.7. Western Blot Assay

The myocardial tissue was cut into small pieces, placed in a RIPA lysate containing a protease inhibitor, homogenized, and centrifuged at 12,000 rpm for 20 min at 4°C, and the supernatant was collected and the protein was quantified using the BCA Protein Quantitation Kit (Nanjing Kaiji). Proteins were separated by SDS-polyacrylamide gel electrophoresis and transferred to PVDF membrane for 2 h. The membrane was blocked by 5% skim milk for 1.5 h and washed by TBST for 3 times (5 min each). The membranes were added with GRP78, CHOP, ERO1*α*, ERO1*β*, PDI, *β*-actin, cleaved caspase-3, Bcl-2, and Bax primary antibody (1 : 1000, Abcam, USA) separately, washed by TBST 3 times (5 min each), and added with HRP-labeled secondary antibody (1 : 5000, Santa Cruz, USA), then incubated for 1 h at room temperature. After washing with TBST, the proteins were illuminated by ECL luminescence kit and imaged by a gel imaging system. ImageJ software was used to calculate the gray value, and the ratio of the target band to the internal reference protein band optical density was used as the result for statistical analysis.

### 2.8. Cell Experiment

#### 2.8.1. Cell Culture and H/R Cell Model Preparation

Rat embryonic cardiomyoblast-derived H9c2 cardiomyocytes were purchased from the Cell Bank of Chinese Academy of Sciences (Shanghai, China). H9c2 were incubated with DMEM containing 10% fetal bovine serum in a 37°C, 95% air, and 5% CO2 incubator. For all cell experiments, cells were cultivated at an appropriate density according to the experimental design and grown for 24 h to reach 70–80% confluence.

The H/R cell model was prepared using a modified process [26]. Briefly, H9c2 was cultured in serum-free DMEM and incubated in an anoxic incubator for 6 h. Then the serum-free DMEM was replaced with DMEM containing 10% fetal bovine serum and incubated for 12 h in a 95% air and 5% CO_2_ incubator to mimic reperfusion.

#### 2.8.2. Cell Grouping and Treatment

Cultured H9c2 cardiomyocytes were randomly divided into four groups. In the control group, H9c2 cardiomyocytes were incubated in high glucose DMEM under normal conditions. The H/R group was prepared as described. When using TM to activate ESR, H9c2 cardiomyocytes were incubated with TM (1 *μ*g/mL) for 24 h. In the Dex-treated group (Dex+H/R or Dex+H/R+TM), H9c2 cardiomyocytes were treated with Dex (1 *μ*M) for 12 h before H/R or TM [27].

#### 2.8.3. Western Blotting for Cells

Proteins were isolated from cells, and a significant volume was loaded, electrophoresed, and transferred to PVDF membranes. Then we detected the expression of cleaved caspase-3, Bcl-2, Bax, GRP78, CHOP, ERO1*α*, and *β*-actin; the protocol was the same as in Section 2.7.

### 2.9. Statistical Analysis

All experiments were repeated at least three times independently. Statistical analysis was performed using SPSS 18.0 software. The experimental results were expressed by the mean SEM, and the data were analyzed by variance. The variance analysis of repeated measures was used for comparison within the group. The analysis of variance was used for comparison between groups. When *P* < 0.05, the difference was considered statistically significant.

## 3. Result

### 3.1. DEX Reduces Serum CK-MB and cTnT Levels in Rats

**Figure 1:**
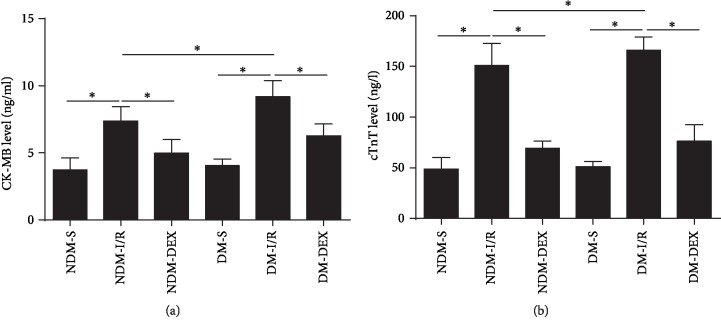
DEX reduces serum CK-MB and cTnT levels in rats. CK-MB and cTnT levels in rat serum were detected by ELISA. (a) CK-MB level. (b) cTnT level. All results are expressed as the mean ± SD. ^∗^*P* < 0.05 between each group.

Creatine Kinase CK-MB is mainly found in cardiomyocytes. When myocardial ischemia occurs, serum CK-MB levels increase rapidly, and the magnitude of the increase directly reflects the degree of myocardial damage [28]. When cardiomyocytes are hypoxic, the free cTnT can be rapidly released into the blood from the cells, and then the cTnT in conjunction with the myocardial structural protein is gradually decomposed and slowly released into the circulating blood; therefore, cTnT levels significantly elevated in the blood [29]. CK-MB and cTnT levels in rat serum were determined by ELISA. The results showed that DEX significantly reduced the levels of CK-MB and cTnT in the serum of NDM-IR and DM-IR rats, and the difference was statistically significant (*P* < 0.05). The difference between the above indicators in the DM-IR group and the NDM-IR group was statistically significant (*P* < 0.05) (Figure 1).

### 3.2. DEX Alleviates Myocardial Tissue Damage in Rats

**Figure 2:**
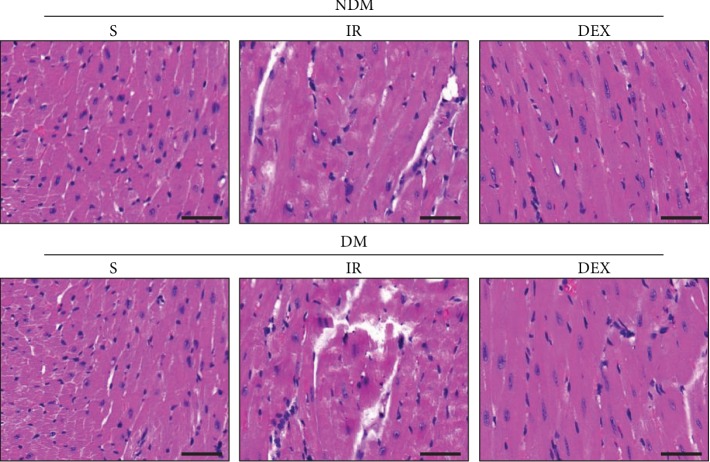
DEX alleviates myocardial tissue damage in rats. The myocardial tissue structure and cell necrosis were observed by HE staining (scale bar = 50 *μ*m).

The myocardial tissue structure and cell necrosis were observed by HE staining. The results showed that the myocardial cells in the NDM-S group had clear horizontal stripes and clear discs and the myocardial microstructure was clear, the arrangement was intact, the staining was uniform, the nucleus and cytoplasm were intact, and there was no cell necrosis. In the NDM-IR and DM-IR groups, myocardial cells are disorderly arranged; many necrotic cells, nucleus lysis and shrinkage, myocardial fiber tear, deep nuclear staining, and myocardial damage in the DM-IR group are more obvious. In the DM-S group, the cell arrangement is relatively uniform, and some necrotic cells are visible. After the DEX treatment, the myocardial cells in the NDM-DEX and DM-DEX groups are completely arranged; the necrotic cells are decreased (Figure 2).

### 3.3. DEX Reduces Myocardial Infarct Size in Rats

**Figure 3:**
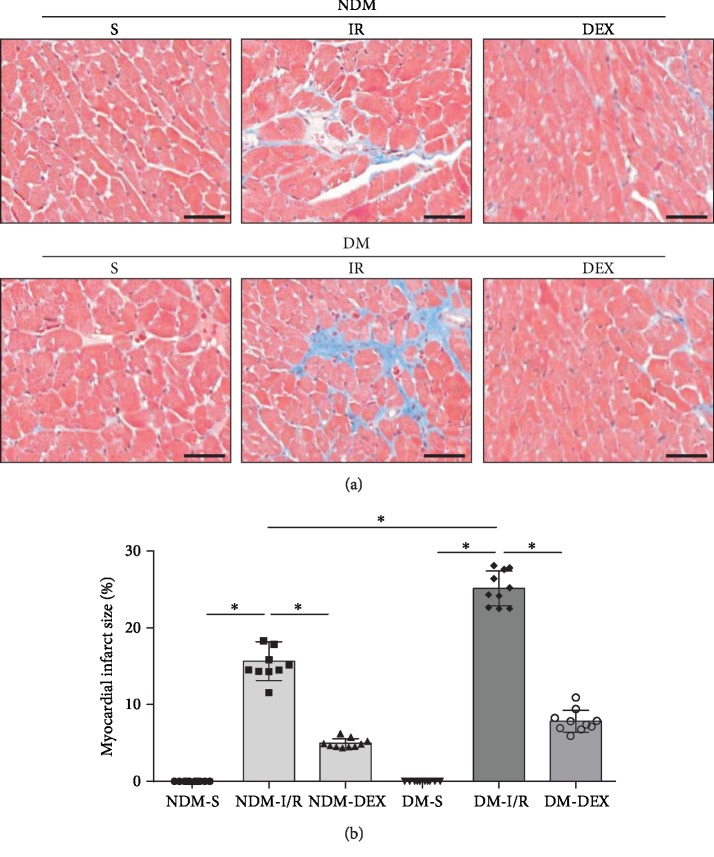
DEX reduces myocardial infarct size in rats. Myocardial infarct size was detected by Masson trichrome staining. (a) Masson staining and myocardial infarction area of rats in each group; the red area represented normal myocardial tissues, while the blue area represented an infarcted myocardium (scale bar = 50 *μ*m). (b) The bar graph of myocardial infarct size: infarct size (%) = (myocardial infarct size/total left ventricular area) × 100%. All results are expressed as the mean ± SD. ^∗^*P* < 0.05 between each group.

To evaluate the effect of DEX on infarct size in rats with myocardial infarction, we used Masson Trichrome Staining to measure myocardial infarct size. The red area represented normal myocardial tissues, while the blue area represented infarcted myocardium. The area of the infarct area is calculated by the ratio of myocardial infarct size/total left ventricular area. The results showed that the area of myocardial infarction in the NDM-IR and DM-IR groups was significantly increased, and the infarct size in the DM-IR group was larger than that in the NDM-IR group. The area of myocardial infarction in the NDM-DEX and DM-DEX groups was significantly reduced (*P* < 0.05) (Figure 3).

### 3.4. DEX Inhibits Cardiomyocyte Apoptosis in Rats

**Figure 4:**
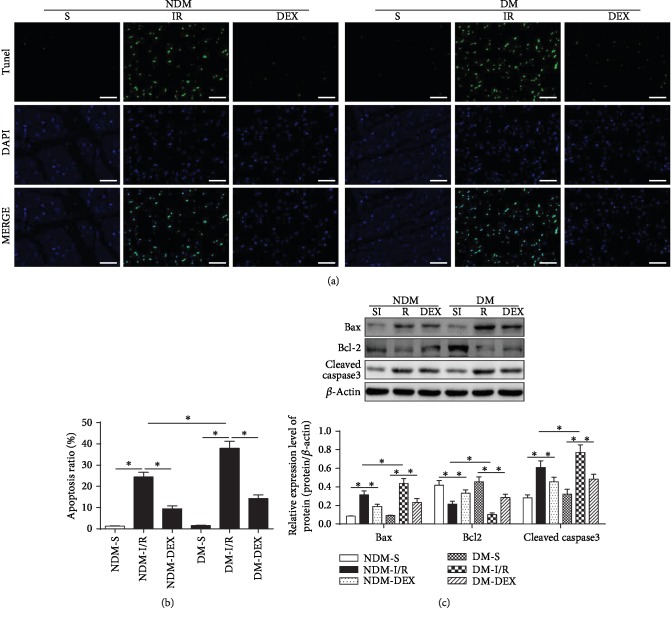
DEX inhibits cardiomyocyte apoptosis. TUNEL staining was used to detect the apoptosis of cardiomyocytes. (a) TUNEL staining (scale bar = 50 *μ*m). (b) The bar graph of the percentage of apoptosis-positive cells. (c) Western blot. All results are expressed as the mean ± SD. ^∗^*P* < 0.05 between each group.

In order to evaluate the apoptosis of cardiomyocytes, we used TUNEL staining for apoptotic cells and used the percentage of apoptosis-positive cells to account for the percentage of total myocardial cells. The results showed that the percentage of apoptosis-positive cells in the NDM-IR and DM-IR groups was significantly increased (*P* < 0.05) and the percentage of apoptosis-positive cells in the DM-IR group was significantly more than those in the NDM-IR group (*P* < 0.05). The percentage of apoptosis-positive cells decreased significantly after DEX intervention, suggesting that DEX intervention can inhibit apoptosis of cardiomyocytes in DM rat combined with MI/IR (*P* < 0.05). The expressions of Bax, cleaved caspase-3, and Bcl-2 protein were detected by western blot. The results showed that the expressions of Bax and cleaved caspase-3 in the NDM-IR and DM-IR groups were significantly increased and the expression of Bcl-2 was significantly decreased (*P* < 0.05). The expressions of Bax and cleaved caspase-3 in the DM-IR group were significantly more than those in the NDM-IR group; the expression of Bcl-2 in the DM-IR group was significantly less than those in the NDM-IR group (*P* < 0.05). The expression of Bax and cleaved caspase-3 decreased significantly, and the expression of Bcl-2 increased significantly after DEX intervention (*P* < 0.05), which is consistent with TUNEL results (Figure 4).

### 3.5. DEX Reduces Myocardial Endoplasmic Reticulum Stress-Related Molecules GRP78 and CHOP in Rats

**Figure 5:**
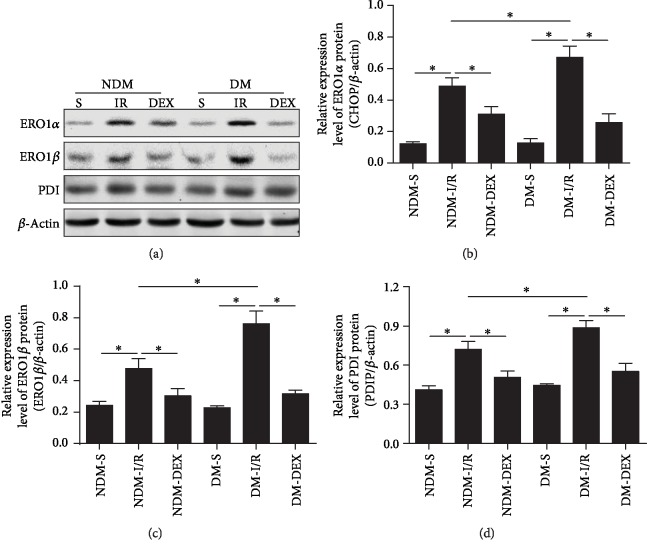
DEX reduces ERO1*α*, ERO1*β*, and PDI protein expression in myocardial tissue. ERO1*α*, ERO1*β*, and PDI protein expressions in the myocardium were detected by western blot. (a) Western blot. (b) The bar graph of ERO1*α* protein expression. (c) The bar graph of ERO1*β* protein expression. (d) The bar graph of PDI protein expression. All results are expressed as the mean ± SD. ^∗^*P* < 0.05 between each group.

**Figure 6:**
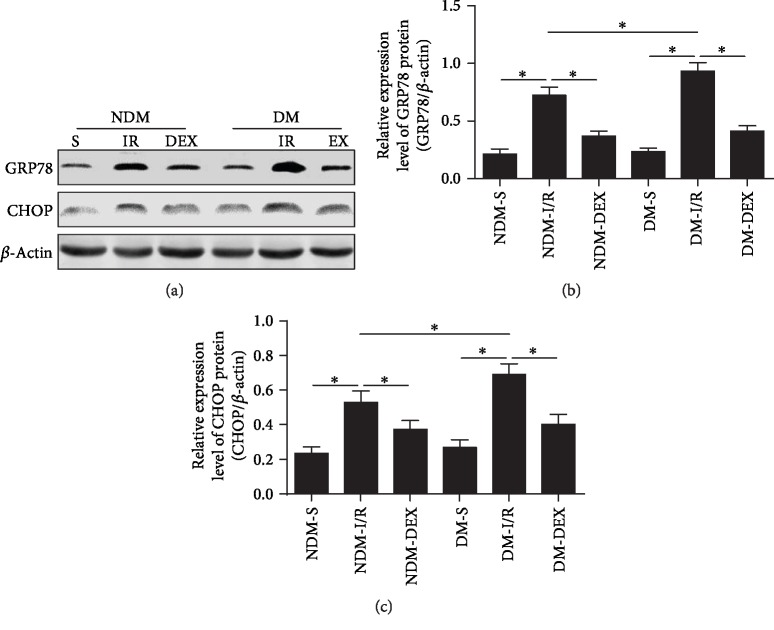
DEX reduces myocardial endoplasmic reticulum stress-related molecules GRP78 and CHOP in rats. GRP78 and CHOP protein expressions in myocardium were detected by western blot. (a) Western blot. (b) The bar graph of GRP78 protein expression. (c) The bar graph of CHOP protein expression. All results are expressed as the mean ± SD. ^∗^*P* < 0.05 between each group.

DM combined with myocardial ischemic disease can lead to myocardial ERS and promote cardiomyocyte apoptosis. GRP78 is a chaperone protein of the endoplasmic reticulum and can participate in the ERS process. The main mechanism is the symptoms of ischemia, hypoxia, and low glucose in tissues and organs, leading to calcium unbalance of the endoplasmic reticulum, resulting in the upregulation of GRP78 expression. GRP78 upregulation activates the ERS-specific apoptotic transcription factor CHOP, which induces apoptosis and promotes disease progression [30, 31]. GRP78 and CHOP in the myocardium were detected by western blot. The results showed that GRP78 and CHOP proteins were significantly elevated in the NDM-IR and DM-IR groups (*P* < 0.05), and GRP78 and CHOP in the DM-IR group were significantly higher than that in the NDM-IR group (Figures 5(a)–5(c), *P* < 0.05), indicating that MI/IR induced myocardial ERS enhancement in rats. The expression of GRP78 and CHOP proteins was significantly decreased after DEX intervention (*P* < 0.05), suggesting that DEX can inhibit ERS from alleviating myocardial ischemia-reperfusion injury in DM rats (Figure 6).

### 3.6. DEX Reduces ERO1*α*, ERO1*β*, and PDI Expression in Myocardial Tissue

ERO1*α*, ERO1*β*, and PDI proteins have a key regulatory role in the production of reactive oxygen in cardiomyocytes and the process of apoptosis [32]. ERO1*α*, ERO1*β*, and PDI were examined by western blot in the myocardial tissue. The results showed that ERO1*α*, ERO1*β*, and PDI in the myocardial tissue of the NDM-IR and DM-IR groups were significantly higher than those of the NDM-S and DM-S groups (*P* < 0.05). In the DM-IR group, the protein expression was higher than that of the NDM-IR group (*P* < 0.05), indicating that MI combined with IR can further upregulate ERO1*α*, ERO1*β*, and PDI expression. After treatment with DEX, the expression levels of ERO1*α*, ERO1*β*, and PDI in the NDM-DEX and DM-DEX groups were significantly downregulated (*P* < 0.05). This result further suggests that DEX can inhibit ERS through attenuating myocardial ischemia-reperfusion injury in DM rats (Figure 5).

### 3.7. Dex Reduces Apoptosis of H9C2 Cells after H/R Injury, Which Was Blocked by ERS Agonist Tunicamycin

**Figure 7:**
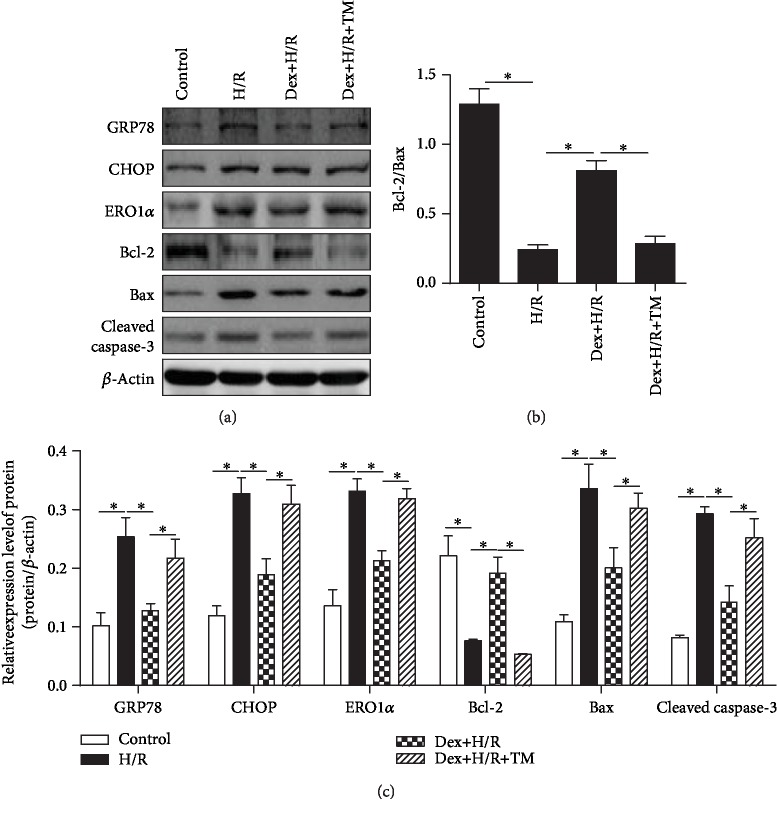
Dex reduces apoptosis of H9C2 cells after H/R injury, which was blocked by ERS agonist tunicamycin. (a) Western blot. (b) The bar graph of Bcl-2/Bax. (c) The bar graph of protein expression. All results are expressed as the mean ± SD. ^∗^*P* < 0.05 between each group.

To verify the effects of Dex on H/R H9c2 cells using an in vitro method of H/R, the apoptosis was detected by examining the expressions of Bax, Bcl-2, and caspase-3, which play an important role in regulating apoptosis. The results showed that compared with the control group, the Bcl-2/Bax ratio was decreased and the expression of cleaved caspase-3 was upregulated in the H/R group (*P* < 0.05). Compared with the H/R group, Bcl-2/Bax was increased, and the expression of cleaved caspase-3 was downregulated in the Dex+H/R group (*P* < 0.05). Compared with the Dex + H/R group, the Bcl-2/Bax ratio was decreased, and the expression of cleaved caspase-3 was upregulated in the Dex+H/R+TM group (*P* < 0.05). These results demonstrated that Dex had an antiapoptotic effect in the H/R cell model, which could be blocked by the ESR agonist. To further detect the relationship between Dex and ERS, the ERS-related protein expressions were examined, including those of GRP78, CHOP, and ERO1*α*. Dex can inhibit the expression of GRP78, CHOP, and ERO1*α*, which are upregulated in the H/R group (*P* < 0.05). The ERS agonist tunicamycin could increase the expression of GRP78, CHOP, and ERO1*α* in the Dex+H/R+TM group compared to the Dex+H/R group; tunicamycin also blocked the effect of Dex on apoptosis of H/R cell (Figure 7).

## 4. Discussion

In recent years, with the aging of the population and changes in lifestyle, the incidence of acute myocardial ischemia has rapidly increased, becoming one of the major diseases that seriously threaten people's health. The prevalence of DM is also increasing year by year, and DM is the main risk factor for acute myocardial ischemia. The incidence of acute myocardial ischemia in patients with DM is significantly higher than that in non-DM patients [33]. Although some progress has been made in the molecular mechanism and treatment in DM aggravating myocardial ischemia, we still need in-depth research. In this study, the model of myocardial ischemia-reperfusion injury in DM rats was found to be significantly greater than those in the NDM-IR and DM-IR groups. The pathological results also showed that the DM-IR group had myocardial ischemia. The degree of histopathological damage was also worse than that of the NDM-IR group, indicating that the myocardial injury was more serious in rats with DM complicated with myocardial ischemia-reperfusion injury.

Many studies have shown that sedative analgesia or anesthesia before reperfusion can reduce myocardial ischemia-reperfusion injury, but DM can eliminate the myocardial protection produced by a variety of drugs (opioids, inhaled anesthetics, etc.) [34–36]. Studies have shown that the extracellular environment such as high glucose, high insulin, and insulin resistance of cardiomyocytes in patients with myocardial ischemia is an important cause of poor prognosis [37]. Therefore, it is of great theoretical and clinical significance in seeking to restore or reconstruct the effective method of DM myocardial sensitivity to posttreatment of therapeutic drugs, thereby improving the ability of DM myocardium to resist ischemia-reperfusion injury. Therefore, the myocardial protection of therapeutic drugs was studied under the pathological model of DM.

DEX is a highly effective and highly selective *α*2 receptor agonist. Due to its positive effects such as analgesia, sedation, and anti-inflammatory response, DEX is widely used in clinical practice as an anesthetic adjuvant. The study also found the protective effect of DEX on vital organs [16]. DEX can be safely used in patients undergoing cardiac surgery, reducing the use of opioid analgesics and sedatives and improving hemodynamic stability in patients [38, 39]. And studies have shown that DEX has a good effect in the treatment of tissue ischemia-reperfusion injury, improving myocardial ischemia-reperfusion injury in DM patients by regulating the GSK-3*β* signaling pathway [40]. DEX can also regulate MAPK and TLR4 signals to reduce the inflammatory response and oxidative stress during cerebral ischemia-reperfusion injury [19, 41], which also alleviate renal ischemia-reperfusion injury in rats [42]. This study found that DEX pretreatment can significantly reduce myocardial ischemic infarct size and pathological changes in DM rats. In addition, it can downregulate the expression of acute myocardial injury markers CK-MB and cTnT, suggesting that DEX can alleviate DM rats with myocardial ischemia-reperfusion injury.

Cardiomyocyte apoptosis is one of the important mechanisms of myocardial dysfunction in DM myocardial ischemic diseases. It has been reported that the DM model of cardiomyocyte apoptosis can not only expand the infarct size but also further affect myocardial cell loss and myocardial remodeling [43–45]. The Bcl-2 family plays an important role in the process of apoptosis. The Bcl-2 family has two representative members: Bax, which promotes the apoptosis gene, and Bcl-2, which inhibits the overexpression of Bcl-2. Overexpression of Bcl-2 can block cell apoptosis, protecting the cardiomyocytes, while the effect of Bax does the opposite [46]. This study found that the number of apoptotic cells in myocardial tissue increased significantly after MI/IR and the number of apoptosis in the DM-IR group was higher than that in the NDM-IR group and the level of antiapoptotic Bcl-2 protein was significantly lower than that in the NDM-IR group. The level of apoptotic Bax protein was significantly higher than that of the NDM-IR group. The above results indicated that the apoptosis of DM with myocardial ischemia-reperfusion injury was more serious. After DEX pretreatment, the apoptosis of cardiomyocytes was significantly improved, indicating that DEX can inhibit cardiomyocyte apoptosis in DM rats with myocardial ischemia-reperfusion injury. These results were further proved in H/R H9c2 cells; Dex was showed to inhibit apoptosis of H9C2 cells after H/R injury, which was blocked by ERS agonist tunicamycin.

Endoplasmic reticulum stress plays an important role in the apoptosis of animal models of DM and myocardial ischemia. When cells encounter various stimuli, the homeostasis of the endoplasmic reticulum is imbalanced, leading to the accumulation of unfolded or misfolded proteins in the cells, resulting in endoplasmic reticulum stress [47]. In the early stage, a certain degree of ERS is conducive to the self-repair of damaged cells, and if the stress is too strong or persists, it will induce the activation of the apoptotic signaling pathway and finally induce cell death.

ERS mediates apoptosis through three main pathways: activation transcription of CHOP/GADD153 gene, activation pathway of C-Jun amino acid kinase (JNK), and activation pathway of caspase-12 [48]. CHOP is an ERS-specific nuclear transcription factor, which acts as an endoplasmic reticulum-associated proapoptotic protein. Under normal physiological conditions, its expression level is extremely low, but it is induced to be expressed in a large amount when ERS occurs, and it is transferred to the nucleus to regulate the expression of related genes inducing apoptosis. GRP78 is a calcium ion-binding molecular chaperone located in the endoplasmic reticulum. It has been reported that when cells undergo endoplasmic reticulum stress, a large amount of misfolded or unfolded protein accumulates in the cell; GRP78 and endoplasmic reticulum cross. Membrane-associated sensor protein separation leads to the activation of downstream CHOP-associated apoptotic signaling pathways [49, 50]. Endoplasmic reticulum oxidoreductase-1 (Ero1) has two isoforms, Ero1*α* and Ero1*β*, which are important causes of intracellular ROS production. Excessive ROS production will destroy the intracellular redox state and induce oxidation, ROS, and ERS promoting apoptosis. CHOP can activate Ero1*α*, catalyze the reoxidation of protein disulfide isomerase (PDI), and ultimately regulate apoptosis. CHOP also upregulates the proapoptotic gene Bax and downregulates the antiapoptotic gene Bcl-2, resulting in the conformation change of Bax/Bak on the endoplasmic reticulum, causing the destruction of the endoplasmic reticulum membrane and Ca^2+^ efflux, which further mediates apoptosis [32, 51, 52].

In this study, we examined the endoplasmic reticulum stress-related proteins GRP78, CHOP, Ero1*α*, Ero1*β*, and PDI in myocardial tissue. It was found that DM can significantly aggravate myocardial ERS in rats with myocardial ischemia and pretreatment of rat myocardial tissue with DEX. The levels of GRP78, CHOP, Ero1*α*, Ero1*β*, and PDI were significantly lower than those in the NDM-IR and DM-IR groups, indicating that DEX can significantly inhibit the CHOP pathway and alleviate ERS levels in rat myocardial tissue.

In summary, DEX can alleviate MI/IR injury possibly alleviating cardiomyocyte apoptosis by mitigating myocardial endoplasmic reticulum stress in DM rat MI/IR rats, thereby alleviating myocardial injury. This finding provides new evidence for the cardioprotective effects of DEX and provides new potential candidates for patients with DM ischemic heart disease.

## Data Availability

The data sets used and analyzed during the current study are available from the corresponding author upon reasonable request.
